# Ovine Herpesvirus 2 Glycoprotein B Complementation Restores Infectivity to a Bovine Herpesvirus 4 gB-Null Mutant

**DOI:** 10.3390/pathogens13030219

**Published:** 2024-03-01

**Authors:** Daniela D. Moré, Katherine N. Baker, Smriti Shringi, Reginaldo G. Bastos, Donal O’Toole, Gaetano Donofrio, Cristina W. Cunha

**Affiliations:** 1 Animal Disease Research Unit, Agricultural Research Service, United States Department of Agriculture, Pullman, WA 99164, USA; katherine.baker@usda.gov (K.N.B.); reginaldo.bastos@usda.gov (R.G.B.); cristina.cunha@usda.gov (C.W.C.); 2 Department of Veterinary Microbiology and Pathology, Washington State University, Pullman, WA 99164, USA; smriti.shringi@ttu.edu; 3 Department of Animal Science, University of Wyoming, Laramie, WY 82070, USA; dot@uwyo.edu; 4 Department of Medical-Veterinary Science, University of Parma, 43126 Parma, Italy; gaetano.donofrio@unipr.it

**Keywords:** viral-vectored vaccine, ovine herpesvirus-2, bovine herpesvirus-4, gammaherpesvirus, glycoprotein B, malignant catarrhal fever, rabbit model

## Abstract

Ovine herpesvirus 2 (OvHV-2) and bovine herpesvirus 4 (BoHV-4) are gamma herpesviruses that belong to the genera *Macavirus* and *Rhadinovirus*, respectively. As with all herpesviruses, both OvHV-2 and BoHV-4 express glycoprotein B (gB), which plays an essential role in the infection of host cells. In that context, it has been demonstrated that a BoHV-4 gB-null mutant is unable to infect host cells. In this study, we used homologous recombination to insert OvHV-2 ORF 8, encoding gB, into the BoHV-4 gB-null mutant genome, creating a chimeric BoHV-4 virus carrying and expressing OvHV-2 gB (BoHV-4∆gB/OvHV-2-gB) that was infectious and able to replicate in vitro. We then evaluated BoHV-4∆gB/OvHV-2-gB as a potential vaccine candidate for sheep-associated malignant catarrhal fever (SA-MCF), a fatal disease of ungulates caused by OvHV-2. Using rabbits as a laboratory model for MCF, we assessed the safety, immunogenicity, and efficacy of BoHV-4∆gB/OvHV-2-gB in an immunization/challenge trial. The results showed that while BoHV-4∆gB/OvHV-2-gB was safe and induced OvHV-2 gB-specific humoral immune responses, immunization conferred only 28.5% protection upon challenge with OvHV-2. Therefore, future studies should focus on alternative strategies to express OvHV-2 proteins to develop an effective vaccine against SA-MCF.

## 1. Introduction

Ovine herpesvirus 2 (OvHV-2) and bovine herpesvirus 4 (BoHV-4) are members of the subfamily Gammaherpesvirinae, classified in the genera Macavirus and Rhadinovirus, respectively [1]. The global dissemination of both viruses within their respective natural hosts underscores the widespread distribution of these viruses on a global scale, exhibiting distinct genomic characteristics and pathogenic potential. BoHV-4 can infect a variety of ruminants with limited pathogenicity as a primary agent, while OvHV-2 is carried asymptomatically by sheep. However, OvHV-2 can cause sheep-associated malignant catarrhal fever (SA-MCF) an often-fatal disease, when transmitted to susceptible species, including cattle, bison, and deer [2,3,4].

As a common feature of all herpesviruses, OvHV-2 and BoHV-4 utilize envelope glycoproteins in a variety of processes during viral entry, assembly, and egress from host cells [5]. Glycoprotein B (gB) is one of the core envelope glycoproteins of herpesviruses and has been shown to be essential for fusion of the viral envelope with the host plasma membrane during viral entry into the host cell [6,7]. Thus, mutations and/or deletions in the gB of herpesviruses affect virus viability, resulting in viral particles that are unable to complete fusion and infect host cells [8,9,10].

The protein structure of gB is relatively conserved across various herpesviruses, with demonstrated functional homology through studies involving mutant viruses and complementation studies. While gB cross-complementation has been demonstrated, it is not always reciprocal or feasible. Successful complementation of gB was achieved between herpes simplex virus 1 (HSV-1) and bovine herpesvirus 1 (BHV-1) and a pseudorabies virus, and between alcelaphine herpesvirus 1 (AlHV-1) and OvHV-2 [8,11,12]. Conversely, complementation failed in other cases, including the substitution of gB in a BoHV-4 gB-null mutant by a homologous glycoprotein from vesicular stomatitis virus (VSV) [13,14,15]. Additionally, viral glycoproteins, including gB, have been considered as vaccine targets for various herpesviruses [16]. Thus, exploring heterologous protein expression and functional complementation among viral glycoproteins offers a potential avenue for designing viral-vectored vaccines, wherein a non-pathogenic virus could serve as the carrier for the vaccine target.

It has been shown that treatment of OvHV-2 with anti-OvHV-2-gB-specific antibodies renders the virus unable to infect cells and cause disease [17]. Therefore, OvHV-2 gB has been investigated as a potential target for a SA-MCF vaccine. Considering the promising results obtained for several vaccine candidates using BoHV-4 as a vaccine vector [18,19,20,21,22,23], we previously engineered a recombinant BoHV-4 to express OvHV-2 gB along with its own gB and tested it as a vaccine candidate against SA-MCF, using rabbits as a disease model [24]. Despite the production of OvHV-2 gB-specific immune responses in the vaccinated animals, only 43% of them were protected from SA-MCF upon challenge with OvHV-2 [24]. It is plausible that the low protection rates observed may be attributed to the possibility that the BoHV-4-gB/OvHV-2-gB chimeric vector predominantly expressed and presented BoHV-4 gB rather than OvHV-2 gB on its surface. This differential expression may have led to a diminished immune response to OvHV-2 gB, contributing to the observed reduction in protection rates. In an attempt to further enhance efficacy, we sought to develop a new strategy to obtain a novel chimeric virus to express exclusively OvHV-2 gB as an essential component for viral infection.

In this work, we successfully integrated OvHV-2 gB into a BoHV-4 gB-null viral genome. The chimeric virus expressed OvHV-2 gB and was infectious for mammalian cells in vitro. Once virus viability was confirmed, we evaluated its potential as an SA-MCF viral-vectored vaccine in a laboratory animal model. Although only a 28.5% protection rate was observed, implications of the results for ongoing efforts to develop an SA-MCF vaccine are discussed.

## 2. Materials and Methods

### 2.1. Construction and In Vitro Evaluation of the BoHV-4∆gB/OvHV-2-gB Mutant

#### 2.1.1. Homologous Recombination

**Figure 1:**
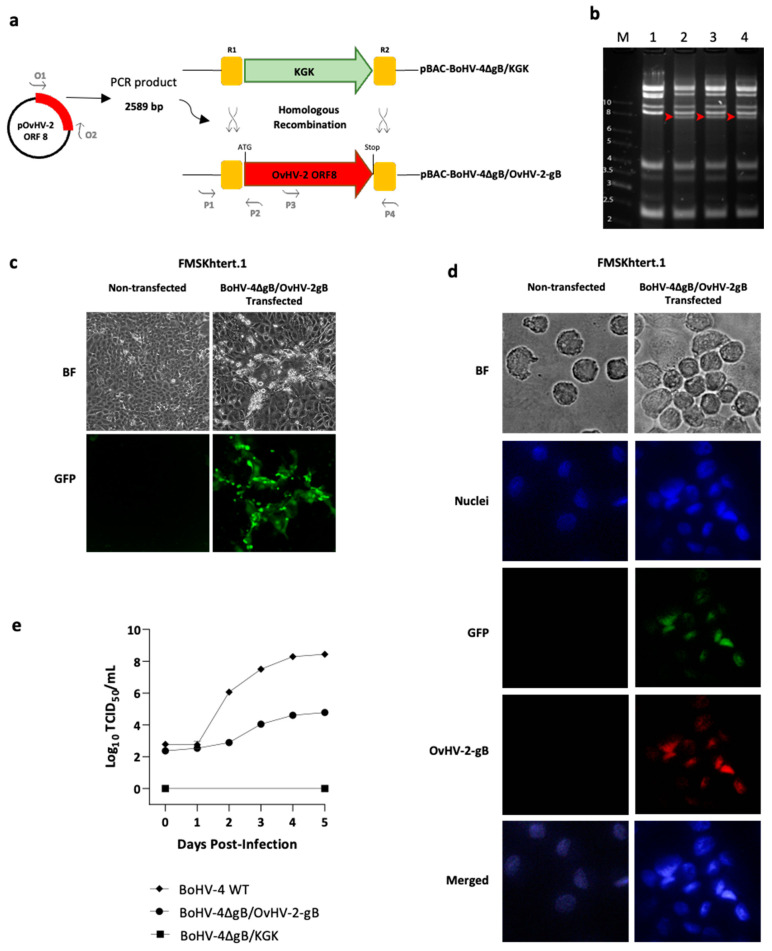
Production and evaluation of the BoHV-4∆gB/OvHV-2-gB chimera. (**a**) Schematic representation of the construction of the recombinant pBAC-BoHV-4∆gB/OvHV-2-gB, where homologous recombination was used to replace the KGK cassette in pBAC-BoHV-4∆gB/KGK by the OvHV-2 ORF8, encoding glycoprotein B (gB). (**b**) Overall integrity of BAC genomes was assessed by EcoRI digestion of pBAC-BoHV-4∆gB-KGK (lane 1) and pBAC-BoHV-4∆gB/OvHV-2-gB DNA (lanes 2, 3, and 4, three independent clones); red-headed arrows show bands that were expected to differ in the newly constructed BAC compared to the parental BAC; M indicates molecular marker (kb). (**c**) Virus reconstitution and spread were confirmed on FMSKhtert.1 cells transfected with pBAC-BoHV-4∆gB/OvHV-2-gB and cultured for five days; green plaques, resulting from the expression of the green fluorescent protein encoded by the BAC cassette, indicate virus reconstitution, replication, and cell-to-cell spread. No plaques or green fluorescence were observed in non-transfected cells. (**d**) OvHV-2 gB expression was demonstrated by immunofluorescence assay using FMSKhtert.1 cells transfected with BoHV-4∆gB/OvHV-2-gB (GFP, green channel) and treated with an OvHV-2-gB-specific monoclonal antibody labeled with Alexa FluorTM 594 (OvHV-2 gB, red channel); BF: bright field; DAPI: 4,6-diamidino-2-phenylindole (nuclei, blue channel); merged: blue, green, and red channels. Non-transfected cells were used as a negative control for GFP and OvHV-2 gB detection. (**e**) Virus growth kinetics of BoHV-4∆gB/OvHV-2-gB, the parental pBAC-BoHV-4∆gB/KGK, and a wild type-BoHV-4 in FMSKhtert.1 cells were evaluated using plaque assay titration.

To generate a recombinant BoHV-4 virus that expresses OvHV-2 gB instead of its own gB, a recombineering technique [25] was used on a BoHV-4 null-gB, previously cloned as a bacterial artificial chromosome (BAC), pBAC-BoHV-4ΔgB-KGK [13]. pBAC-BoHV-4ΔgB-KGK has the gB encoding gene (ORF8) disrupted by the insertion of a kanamycin resistance and galK gene cassette (KGK) in its locus. In the study reported here, we replaced the KGK cassette in pBAC-BoHV-4ΔgB-KGK with the OvHV-2 gB-encoding gene and obtained the pBAC-BoHV-4∆gB/OvHV-2-gB mutant. A schematic representation of the recombineering step is shown in Figure 1a. Briefly, the full-length OvHV-2 ORF8 DNA fragment, flanked by R1 and R2 arms, was amplified by PCR using oligos O1 and O2 as primers (Table S1) and a plasmid containing a mammalian codon-optimized sequence of OvHV-2 ORF8 [17] as a template. The OvHV-2 ORF8 fragment was transformed into SW102 *E. coli,* containing the pBAC-BoHV-4 ∆gB/KGK, generating the pBAC-BoHV-4∆gB/OvHV-2-gB. Negative selection was performed on plates containing minimal medium and 2-deoxygalactose with glycerol, where clones containing the galK gene are unable to grow. The selection was confirmed by culturing selected clones in the presence of kanamycin, where clones that had the kanamycin gene replaced by OvHV-2 ORF8 would not grow. DNA from selected pBAC-BoHV-4∆gB/OvHV-2-gB *E. coli* clones was isolated using NucleoBond^®^ BAC 100 (Clontech Laboratories, Inc., Mountain View, CA, USA). Correct recombination events were verified by PCR using primers covering both left and right recombination junctions (Table S1, P1 to P4), followed by sequencing of amplicons. The integrity of the pBAC-BoHV-4∆gB/OvHV-2-gB genome was assessed by comparing the EcoRI digestion profile relative to the parental strain.

#### 2.1.2. Recombinant Virus Reconstitution

The ability of pBAC-BoHV-4∆gB/OvHV-2-gB to produce infectious viral particles was assessed by plaque formation in mammalian cells following BAC DNA transfection. Briefly, immortalized fetal mouflon sheep kidney (FMSKhtert.1) cells were cultured in 6-well plates using complete Dulbecco’s Modified Eagle Medium, high glucose, GlutaMAX™ Supplement, pyruvate (ThermoFisher Scientific, Waltham, MA USA), containing 10% FBS, 100 U/mL penicillin, 100 µg/mL streptomycin, and 1 µg/mL amphotericin B (c-DMEM) at 37 °C and 5% CO_2_ until 70–80% confluency. The cells were transfected with BAC DNA (1.2 µg/well of pBAC-BoHV-4∆gB/OvHV-2-gB) using Attractene Transfection Reagent (Qiagen, Valencia, CA, USA), in accordance with the manufacturer’s recommendations. Transfection was confirmed and monitored by the visualization of plaques expressing the green fluorescence protein (GFP) encoded by the BAC cassette, using fluorescence microscopy. The Personal Automated Lab Assistant (PAULA, Leica Microsystems, Wetzlar, Germany) was used to record and image cell cultures. Reconstituted virions were harvested at five days post-transfection following three freeze–thaw cycles. The reconstituted chimeric virus is referred to here as BoHV-4∆gB/OvHV-2-gB. The loxP-flanked BAC cassette in BoHV-4∆gB/OvHV-2-gB was excised following three passages in FMSKhtert.1/Cre cells, as previously demonstrated [8]. Excision of the BAC cassette was monitored and confirmed by the loss of GFP expression in viral plaques using fluorescence microscopy.

#### 2.1.3. Virus Propagation and Titration

For viral propagation, confluent monolayers of FMSKhtert.1 cells were infected with virus for 4–6 h, split, and cultured in c-DMEM until cytopathic effect reached about 90%. The virus was harvested after three freeze–thaw cycles, clarified by centrifugation (3000× *g*, 30 min at 4 °C), and stored at −80 °C until use. The titer of virus stocks was determined as 50% tissue culture infectious dose (TCID_50_) following a limiting dilution assay using standard methods. Briefly, the virus stock was serially diluted in c-DMEM, and each viral dilution (100 µL/well) was added to 80–90% confluent FMSKhtert.1 cells in 96-well plates and cultured for eight days. Eight replicates of each viral dilution were used per assay. The presence or absence of plaques in each well was verified by microscopic examination, following standard 10% formaldehyde fixation and 0.1% crystal violet staining.

#### 2.1.4. Viral Growth Curves

FMSKhtert.1 cells were infected with wild-type BoHV-4 or BoHV-4∆gB/OvHV-2-gB at an MOI of 0.1 in triplicate. Cells and culture supernatants were collected at 24 h intervals for five days and transferred to −80 °C. Following three freeze–thaw cycles, infectious particles in each preparation were titrated, as described above. Cells transfected with DNA from the parental BAC (pBAC-BoHV-4∆gB/KGK) and harvested at five days post transfection were used as a reference.

#### 2.1.5. OvHV-2 gB Expression

The expression of OvHV-2 gB was assessed by immunofluorescence using FMSKhtert.1 cells transfected with BoHV-4∆gB/OvHV-2-gB. At five days post-infection, cells were washed three times in PBS, harvested using 10 mM EDTA, deposited onto a glass slide, and immediately fixed (3:1 ratio of methanol and acetone) at −20 °C for 5 min. For immunofluorescence, cells were probed with anti-OvHV-2 gB F1.2 monoclonal antibody as the primary antibody [24], and an Alexa Fluor^®^ 594 goat anti-mouse IgG (H+L) as the secondary antibody (Invitrogen, Carlsbad, CA, USA). Slides were mounted with ProLong™ Diamond Antifade Mountant with DAPI (Invitrogen, Carlsbad, CA, USA) and examined using a fluorescence microscope. Images were obtained and analyzed using LAX software (version 3.6.1.26246, Leica Microsystems, Wetzlar, Germany).

### 2.2. Evaluation of BoHV-4∆gB/OvHV-2-gB as an SA-MCF Vaccine

#### 2.2.1. Immunization, Challenge, and Animal Monitoring

The immunogenicity and efficacy of the BoHV-4∆gB/OvHV-2-gB mutant as a vaccine candidate for SA-MCF were tested using rabbits as a laboratory animal model. Animal housing, maintenance, and experimentation followed Washington State University Institutional Animal Care and Use Committee-approved protocols (WSU IACUC ASAF #6364). The virus used for immunizations had the BAC cassette removed in FMSKhtert.1/Cre cells. Fourteen rabbits (n = 7/group) were immunized intravenously with BoVH-4∆gB/OvHV-2-gB (10^5^ TCID_50_ in 1 mL) obtained from infected FMSKhtert.1 cells or medium from uninfected cells (1 mL). One-prime-two-booster immunizations were performed at three-week intervals. Three weeks after the last immunization, all rabbits were challenged intranasally with a lethal dose of OvHV-2 (10^6^ OvHV-2 genome copies) delivered by nebulization, as previously described [26].

Rabbits were monitored daily for adverse effects of the immunizations and following the challenge, for clinical signs of MCF. Blood samples were collected throughout the experiment to assess antibody responses and viral loads. MCF-affected animals were euthanized within 48 h once sustained rectal temperature higher than 40 °C was observed. At 139 days post-prime immunization (DPI), the experiment was terminated, and all remaining rabbits were euthanized and necropsied. Lung tissues were collected and processed for pathological analysis and viral load quantification. Histology was performed blindly by a pathologist, and results were reported as no visible lesion (NVL); or mild (+), moderate (++), or severe (+++) lesions. Vaccine efficacy was estimated by calculating the percentage of animals in each group that were protected or developed SA-MCF following challenge.

#### 2.2.2. Viral DNA Quantification

OvHV-2 levels in blood and/or tissues were assessed by quantitative PCR (qPCR) targeting OvHV-2 ORF 75, as previously described [27]. PCR results were analyzed using CFX96 Manager software (version 3.1, BioRad, Hercules, CA, USA) and reported as OvHV-2 genome copies/50 ng of total DNA.

#### 2.2.3. Antibody Detection

Plasma from vaccinated and mock-immunized rabbits were tested by ELISA for quantification of anti-BoHV-4 and anti-OvHV-2-gB antibodies. For anti-BoHV-4 antibody detection, the samples were tested at a 1:100 dilution using a commercial kit (Bio-X Diagnostics Monoscreen AbELISA BoHV-4, Rochefort, Belgium), following the manufacturer’s instructions. Results are shown as the 450 nm optical density (OD) of a tested sample to the BoHV-4 antigens after subtracting reactivity to the no antigen control. For anti-OvHV-2-gB detection, 96-well plates were coated with protein lysate from HEK293T cells transfected with a plasmid expressing OvHV-2 gB, as described previously [17]. Samples were tested at a 1:400 dilution. Anti-rabbit IgG/HRP and TMB were used for detection. Optical densities (OD) were read at 450 nm, and results are shown as a ratio of the tested sample OD (S) divided by the background (blank OD, B).

#### 2.2.4. Virus Neutralization

OvHV-2 gB neutralizing antibodies were measured by in vitro neutralization assay using a recombinant alcelaphine herpesvirus 1 (AlHV-1∆gB/OvHV-2-gB), which expresses OvHV-2 gB and can be neutralized by anti-OvHV-2-gB antibodies. This assay has been previously optimized for the measurement of OvHV-2 gB-dependent neutralizing activity [8]. Briefly, 1:32 diluted plasma or 1:4 diluted nasal secretions (NS) and bronchoalveolar lavages (BALs) were treated at 56 °C for 10 min to inactivate the complement, mixed 1:2 with 10^2^ TCID_50_ AlHV-1∆gB/OvHV-2-gB in triplicate, and incubated for 1 h at 37 °C. The mixture was then transferred to FMSKhtert.1 cell cultures and kept for five days, after which cells were fixed and plaques were counted. The average number of plaques from each condition was used to calculate the percentage of inhibition using the following formula: % inhibition = 100 − (X × 100/Max), where X is the average number of plaques in the tested sample and Max is the number of plaques in the virus only control.

#### 2.2.5. Statistical Analysis

Viral growth and OvHV-2-specific humoral responses over time were analyzed using two-way (time/treatment) repeated measures ANOVA (mixed-effect model) with 95% confidence intervals, followed by Sidak’s multiple comparison post-test. Neutralizing antibody activity was compared using an unpaired *t*-test with a = 0.05. *p*-values lower than 0.05 were considered statistically significant. Protection rates were compared by Fisher’s exact test, which has 80% power to detect a relative difference of 47% between vaccinated and mock groups, with a significance level of 0.05 (one-tailed) when 100% of animals in the mock group develop MCF (GraphPad StatMate 2.0.0, Boston, MA, USA). GraphPad Prism 9 software (Boston, MA, USA) was used for statistical analyses and graph design.

## 3. Results

### 3.1. Virus Construction and Complementation

In this study, we inserted the gB encoding gene from OvHV-2 into a non-infectious BoHV-4 gB-null strain and tested the ability of the OvHV-2 gB to reconstitute a viable virus that can infect and replicate in mammalian cells. The construction and in vitro characterization of the new chimeric virus are illustrated in Figure 1.

Following amplification of the full-length OvHV-2 ORF8 DNA sequence flanked by the R1 and R2 sites, homologous recombination was used to replace the KGK cassette from pBAC-BoHV-4∆gB/KGK with the OvHV-2 ORF8 fragment. This procedure resulted in the pBAC-BoHV-4∆gB/OvHV-2-gB chimera (Figure 1a), which was designed to express the full-length OvHV-2 gB under the control of the original BoHV-4 promoter. Restriction digestion patterns of DNA, extracted from selected *E. coli* colonies containing either pBAC-BoHV-4∆gB/OvHV-2-gB or the parental virus BAC, showed the presence of the additional bands expected from recombination and verified the overall integrity of the pBAC-BoHV-4∆gB/OvHV-2-gB genome (Figure 1b). Correct recombination events were also confirmed by sequencing the regions where recombination was expected to occur.

Viability of the newly constructed chimeric virus was assessed by transfecting FMSKhtert.1 cells with pBAC-BoHV-4∆gB/OvHV-2-gB DNA and monitoring plaque formation. Plaques were visualized with both bright field and fluorescence microscopy, indicating that virions reconstituted from the BAC were able to infect and spread in the cell monolayer (Figure 1c). These results confirm that the BoHV-4∆gB/OvHV-2-gB was infectious.

The expression of OvHV-2 gB by the chimeric virus was verified by immunofluorescence in pBAC-BoHV-4∆gB/OvHV-2-gB transfected FMSKhtert.1 cells using a OvHV-2 gB-specific monoclonal antibody. Red staining resulting from the detection of OvHV-2 gB was visualized in transfected cells, while no staining was observed in non-transfected cells (Figure 1d). Colocalization of OvHV-2 gB and GFP from the BAC cassette and cell nucleus confirms BoHV-4∆gB/OvHV-2-gB infection and expression of OvHV-2 gB.

To evaluate the replication kinetics of the new chimeric virus, its growth curve was compared with a wild type BoHV-4 (Figure 1e). Although cells were infected with a similar amount of virus, significantly lower titers of BoHV-4∆gB/OvHV-2-gB (10^4.8^) were observed when compared to the wild type (10^8.4^) from two to five days post-infection (*p* < 0.0001). As expected, no replication was observed in cells transfected with the parental BAC, pBAC-BoHV-4∆gB/KGK, up to five days post-transfection.

Overall, these results demonstrated that OvHV-2 ORF 8 replaced the KGK cassette in the non-viable pBAC-BoHV-4∆gB/KGK and that the resultant BAC reconstituted infectious viral particles, confirming that OvHV-2 gB can restore infectivity to the BoHV-4-null-gB virus in vitro.

### 3.2. Evaluation of BoHV-4/OvHV-2-gB as a Potential SA-MCF Vaccine Candidate

Once we obtained the chimeric BoHV-4∆gB/OvHV-2-gB virus, it was of interest to evaluate it in vivo to assess its potential as an SA-MCF vaccine. Thus, we used a well-established rabbit model of SA-MCF to verify the immunogenicity and efficacy of this chimera in a vaccine/challenge trial.

**Figure 2:**
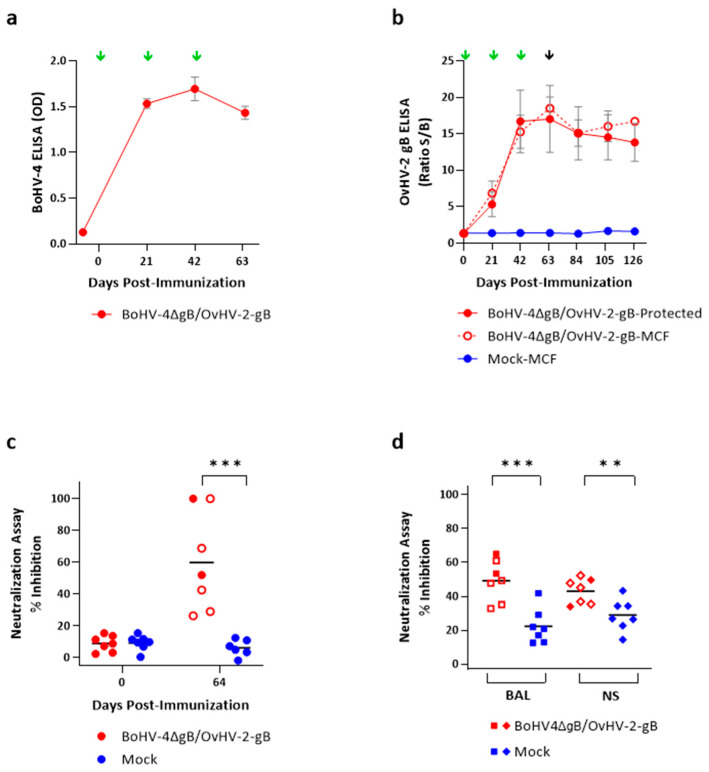
Antibody production in rabbits immunized with BoHV-4∆gB/OvHV-2-gB or with a mock inoculum and challenged with OvHV-2. (**a**) BoHV-4 antibody response in plasma, as measured by a BoHV-4 commercial ELISA. (**b**) OvHV-2-gB antibody response in plasma as quantified by an OvHV-2 gB-specific ELISA. In panels (**a**,**b**), green arrows indicate immunizations times and the black arrow challenge; error bars represent SEM. gB-dependent neutralization of an AlHV-1∆gB/OvHV-2-gB chimeric virus by antibodies in plasma, bronchoalveolar lavage (BAL), and nasal secretions (NS), as measured by in vitro virus neutralization assay, are shown in panels (**c**,**d**), with bars indicating the means. (**c**) Percentage of inhibition using plasma samples obtained prior to immunization, 0 DPI, and on the day of OvHV-2 challenge, 64 DPI. (**d**) Percentage of inhibition using bronchoalveolar lavage (BAL) and nasal secretions’ (NS) terminal samples. In (**b**–**d**), BoHV-4∆gB/OvHV-2-gB-immunized animals that developed MCF following OvHV-2 challenge are represented by open symbols. **, *p* < 0.01, and ***, *p* < 0.001.

Rabbits were immunized by intravenous inoculation with either BoHV-4∆gB/OvHV-2-gB (n = 7) or uninfected cell culture medium as a mock immunization (n = 7) three times at three-week intervals. No adverse effects were observed after inoculations, suggesting that immunizations were safe. All rabbits were challenged with a potentially lethal dose of OvHV-2 at 64 DPI. Five out of seven rabbits that were immunized with BoHV-4∆gB/OvHV-2-gB and all seven rabbits that received the mock immunization developed SA-MCF. All seven rabbits in the BoHV-4∆gB/OvHV-2-gB group seroconverted to BoHV-4 antibodies after prime immunization and maintained high levels of antibodies in plasma until the last test at 64 DPI (Figure 2a).

Immunization with BoHV-4∆gB/OvHV-2-gB also induced systemic OvHV-2 gB-specific humoral responses (Figure 2b), which were significantly higher than with the mock immunization (*p* > 0.0001) starting at 21 DPI. In rabbits immunized with BoHV-4∆gB/OvHV-2-gB, antibody levels in plasma were, on average, 6-fold higher at 21 DPI than the pre-immunization levels, peaked at an 18-fold increase right before OvHV-2 challenge (64 DPI), and remained relatively stable until 126 DPI, the last day tested. In animals that were immunized with BoHV-4∆gB/OvHV-2-gB, there was no significant difference in OvHV-2 gB antibody levels between rabbits that were protected from the disease or that developed MCF following challenge (adjusted *p* = 9975). Animals in the mock group did not seroconvert.

Considering the potential role of neutralizing antibodies in protection, we evaluated plasma (Figure 2c) and BAL and NS (Figure 2d) samples from BoHV-4∆gB/OvHV-2-gB-immunized and mock animals for OvHV-2 gB-specific neutralizing antibodies. As expected, pre-immunization plasma samples from all animals did not neutralize the virus (mean percentage of inhibition, plus/minus standard deviation: BoHV-4∆gB/OvHV-2-gB = 8.9 ± 5.0, Mock = 9.4 ± 4.7; *p* = 0.8631). However, significantly higher OvHV-2 gB-dependent viral neutralization (*p* = 0.0014) was detected at 64 DPI in the plasma of BoHV-4∆gB/OvHV-2-gB-vaccinated animals, when percentages of inhibition ranged from 26.4% to 100% (59.8 ± 30.9), while the mock group remained at basal levels ranging from 0 to 12.5% (6.5 ± 4.3). At 64 DPI, when animals were challenged with OvHV-2, the two rabbits that remained healthy throughout the experiment showed 52% and 100% inhibition, which was within the range of inhibition observed in the five animals that developed MCF (53.4 ± 30.9). We also observed significant differences in neutralizing activity in BAL (*p* = 0.0008) and NS (*p* = 0.0088) between BoHV-4∆gB/OvHV-2-gB and mock groups when the animals were euthanized (Figure 2d). In BAL, average percentages of inhibition of 49.2 ± 12.1 and 22.3 ± 10.3 were measured in the vaccinated and mock groups, respectively, with similar values detected in NS (43.0 ± 7.5 for vaccinated and 28.9 ± 9.4 for mock). The percentages of inhibition for each animal are listed in Table S2.

The efficacy of immunization with BoHV-4∆gB/OvHV-2-gB in protecting animals against MCF was assessed at the end of the study at 74 days post-challenge. As shown in Table 1, only two out of seven BoHV-4∆gB/OvHV-2-gB immunized animals were protected and remained healthy until the experiment termination, whereas the remaining five vaccinated and all seven mock animals developed MCF, which is equivalent to a protection rate of 28.5%, with no significant difference between the groups (*p* = 0.4615).

MCF development was similar in all animals, regardless of immunization status. No difference in the time for detection of OvHV-2 DNA in blood, onset of fever, and viral load in lung tissues were observed among the animals, as summarized in Table 2. Histological lesions compatible with MCF, including inflammation and vasculitis, were observed in all animals that developed disease. The two rabbits immunized with BoHV-4∆gB/OvHV-2-gB that remained healthy after challenge had no detectable OvHV-2 DNA in blood at any time, and there were no histological lesions in any organ examined. Collectively, the results demonstrate that BoHV-4∆gB/OvHV-2-gB immunized animals developed humoral responses to OvHV-2 gB, including neutralizing antibodies, but minimal protection against development of MCF was observed.

## 4. Discussion

Our results show that OvHV-2 gB can complement the BoHV-4 gB-null mutant and restore its infectivity. Visualization of plaques following the transfection of FMSKhtert.1 cells with pBAC-BoHV-4∆gB/OvHV-2-gB and reactivity of an anti-OvHV-2-gB antibody in infected cells confirm that virions that express OvHV-2 gB were reconstituted from the BAC. Although interactions between virus proteins and cells during the entry process in BoHV-4 and OvHV-2 are not completely understood [6,28], core glycoproteins, including gB and the heterodimer gH/gL, are known to work together to promote fusion in herpesviruses [29,30]. Since BoHV-4 can infect cells using OvHV-2 gB, it is likely that BoHV-4 and OvHV-2 gB share common features involved in the cascade of events that result in the virus infection of host cells and, ultimately, cell-to-cell spread. Although the complementation of gB in the non-infectious pBAC-BoHV-4-∆gB-KGK by OvHV-2 ORF 8 enabled viral replication and growth in vitro, significantly lower viral titers and growth kinetics were observed as compared to the wild-type BoHV-4, suggesting that the chimeric virus, albeit infectious, had a replicative deficit. It was out of the scope of the current study to investigate if such a deficit was associated with gB only or if the ORF 8 replacement also affected potential accessory glycoproteins involved in virus entry, and additional investigations are necessary to address these hypotheses.

Following complementation of the BoHV-4 gB-null mutant by OvHV-2 gB, it was of interest to evaluate the BoHV-4-vectored OvHV-2 gB as an SA-MCF vaccine candidate. The development of a vaccine for SA-MCF is a necessity for several livestock industries and zoos, and it is, therefore, a priority for research. We have previously tested another BoHV-4-vectored OvHV-2 gB vaccine candidate in a rabbit model but observed low efficacy in protecting animals from SA-MCF following OvHV-2 challenge [24]. To further enhance efficacy, in this current study, we used an alternative engineering strategy to obtain a BoHV-4 that expresses only OvHV-2 gB. Compared to the previous work, where the viral vector expressed both the BoHV-4 and OvHV-2 gB proteins, the virus used in this study had the BoHV-4 gB gene replaced by the OvHV-2 homolog. Because gB is essential for virus infection, the newly constructed virus was expected to express OvHV-2 gB more efficiently than the previous one and, consequently, be more effective as a vaccine. However, the newly constructed virus failed to enhance efficacy and had reduced protection to SA-MCF in the rabbit model compared to the previous study. Although several factors can affect vaccine efficacy, the replication deficit observed in vitro may have also occurred in vivo and, in part, contributed to the poor performance of BoHV-4∆gB/OvHV-2-gB as a vaccine. In addition, the dose of virus used in each immunization might have been insufficient to induce a protective immune response. Low immunization doses and/or impaired expression of the vaccine target could affect the type and magnitude of immune responses developed after immunization, consequently resulting in low protection rates in vaccinated animals.

Notably, all BoHV-4∆gB/OvHV-2-gB-immunized rabbits seroconverted to both BoHV-4 and OvHV-2 gB and developed high levels of antibodies, confirming the immunogenicity of the chimeric virus. Neutralizing antibody responses were measured in a neutralization assay based on an OvHV-2 gB-expressing virus with a different backbone from the vaccine vector, allowing for the detection of OvHV-2 gB-specific neutralization activity while avoiding anti-vector antibody neutralization responses. OvHV-2 gB-specific neutralizing antibodies were present in plasma at the time of challenge (64 DPI) and in BAL and NS samples collected during necropsy. Significantly higher neutralizing activity was observed following BoHV-4∆gB/OvHV-2-gB immunization compared to mock immunization, suggesting that the vaccine candidate induced mucosal neutralizing antibodies in the respiratory tract as well. However, regardless of the presence of systemic and mucosal humoral responses, similar levels of antibodies and neutralizing activities were observed in BoHV-4∆gB/OvHV-2-gB-immunized rabbits that were protected or developed MCF following challenge. For instance, plasma samples from two rabbits showed 100% inhibition in the neutralization assay, but only one of the animals was protected from MCF following challenge. It is important to note that the assay used to measure neutralizing activity does not result in antibody titration. Therefore, it is possible that two animals showing 100% inhibition in plasma diluted at 1:32 indeed have highly different titers of neutralizing antibodies. Overall, antibody responses obtained in this study are in accordance with previous SA-MCF vaccine trials using BoHV-4 or AlHV-1-vectored OvHV-2 gB vaccine candidates tested in the rabbit model, where the presence of anti-OvHV-2-gB antibodies and protection were also not correlated [24,31]. Taken together, these results suggest that other immune factors, including cellular immunity, are critical for protection against SA-MCF. Although rabbits are a reliable animal model for MCF, tools to better characterize their cellular responses are limited, preventing a complete evaluation of their immune responses. For this reason, in these initial screenings of vaccine candidates, we opted to look only at humoral responses, while more comprehensive analyses of immune responses induced by vaccination will be performed in future studies, when selected candidates are tested in natural hosts, such as cattle and bison.

Interestingly, the absence of OvHV-2 DNA in the blood and tissues of the two animals that did not develop SA-MCF upon challenge suggests that protection was achieved by preventing OvHV-2 infection rather than by a reduction in viral load. This, along with other studies targeting OvHV-2 gB as a vaccine [24,31], confirms that sterile immunity based on OvHV-2 gB can be achieved by vaccination.

It has been demonstrated that previous exposure to natural BoHV-4 infection and the presence of preexisting anti-BoHV-4 antibodies in cattle did not prevent the development of a robust antibody response upon immunization with BoHV-4-vectored vaccine candidate for bovine viral diarrhea virus and bovine herpesvirus 1 [32]. Although the reasons for the sub-optimal rate of protection observed in this study remain unclear, anti-vector immune responses due to homologous booster immunizations might have negatively affected the exposure of the animals to the vaccine target. Therefore, the association between BoHV-4 previous exposure/immunization and vaccine efficacy needs further investigation considering its use as a vaccine vector.

A possible modification to improve the efficacy of the vaccine candidate tested in this study would be the inclusion of additional OvHV-2 glycoproteins, such as gH and gL, which have also shown to induce antibodies capable of blocking OvHV-2 [17]. Different vaccination strategies, such as heterologous immunization protocols, alternative delivery systems, and/or the use of adjuvants as immune modulators and enhancers, should also be considered in future studies.

## 5. Conclusions

OvHV-2 gB can complement a non-infectious BoHV-4 gB-null mutant and restore its infectivity. The resulting BoHV-4∆gB/OvHV-2-gB chimera was tested as an MCF vaccine candidate in a rabbit model, and although it induced OvHV-2-specific immune responses, including neutralizing antibodies, only limited protection (28.5%) against MCF following challenge with OvHV-2 was observed. Future studies should focus on more efficient strategies for expressing OvHV-2 proteins and/or alternative vaccine platforms with the goal of developing an effective vaccine for SA-MCF.

## Figures and Tables

**Table 1:** Protection efficacy against SA-MCF following OvHV-2 challenge in BoHV-4∆gB/OvHV-2-gB or mock-immunized animals.

Group	Immunization	Protected (%)	MCF (%)	Total
Vac	BoHV-4∆gB/OvHV-2-gB	2 (28.57%)	5 (71.43%)	7
Mock	Mock	0 (0.00%)	7 (100.00%)	7
Total		2	12	14

**Table 2:** Parameters associated with the development of SA-MCF in rabbits challenged with OvHV-2.

Rabbit ID	Group	First Detection of OvHV-2 DNA in Blood by qPCR (DPC)	Onset of Fever (DPC)	MCF (DPC)	OvHV-2 Genome Copies in Lungs (qPCR)	Histological Lesion Scores
3060	V	-	-	-	0	NVL
3061	V	-	-	-	0	NVL
3062	V	17	19	20	8210	+
3063	V	53	72	72	10,900	+
3064	V	20	24	26	34,300	++
3065	V	17	20	22	29,500	+
3066	V	24	33	34	47,500	+
**Avg (V)**		**26.2**	**33.6**	**34.8**	**26,082**	
3070	M	26	35	36	27,800	+
3071	M	11	23	24	19,500	+
3072	M	36	49	49	10,800	+
3073	M	17	25	26	16,900	+
3074	M	20	27	27	35,800	+
3075	M	20	27	27	55,600	++
3076	M	17	25	26	25,200	+
**Avg (M)**		**21**	**30.14**	**30.7**	**27,371**	

V, BoHV-4∆gB/OvHV-2-gB immunization; M, mock immunization; qPCR, OvHV-2 quantitative PCR; DPC, days post-OvHV-2 challenge; NVL, no-visualized lesion; +, mild or ++, moderate lesion severity.

## Data Availability

The data that support the findings of this study are available upon reasonable request.

## Supplementary Materials

The following supporting information can be downloaded at: https://www.mdpi.com/article/10.3390/pathogens13030219/s1, Table S1: Oligos/primers used for construction and evaluation of the pBAC-BoHV-4∆gB/OvHV-2-gB.; Table S2: Individual percentages of inhibition in AlHV-1∆gB/OvHV-2-gB neutralization assay.
