# Supplementary material for: Ovine Herpesvirus 2 Glycoprotein B Complementation Restores Infectivity to a Bovine Herpesvirus 4 gB-Null Mutant
Source: Pathogens. 2024 Mar 1;13(3):219. doi: 10.3390/pathogens13030219 (PMC10974308; doi:10.3390/pathogens13030219)
Supplement: Supplementary file 1 [file pathogens-13-00219-s001.zip › pathogens-2819681-supplementary Table S1.pdf]

Table S1. Oligos/primers used for construction and evaluation of the pBAC-BoHV-4ΔgB/OvHV-2-gB.

Oligo/Primer	Sequence (5′-3′)
**O1**	ATGATAACACTGGGTGGATATTTAAAGACTTATATGCCCTTCTTTACCACCACCTACAGTTGACTAGCC ATAAAAAATGGCTTCTCCTACCTCTACCCTGT
**O2**	TTTTAGATATGTATGGGTTAAAAAACTCCATTGCATAGAGACACTCTGCGGGGCAGGGTGTTTGGGGAA TGTACAGACTTTATGTAGAAAGACCAGAGGGAATCTGTCTTCTAAAAAACATATTTTATTTGTTTCCTGG CAGTAAATACACACTGAAAAGGCAGCTTGTTTCACAGGGCAGCAGCAGACTCGGTAGAATC
**P1**	GAGGTCATCTGGCCATCCAT
**P2**	AGCCCTCCTCCTCATTCAG
**P3**	CTGGTGGACTACAAGGAC
**P4**	GCTCCTTTGATGATCCCCCG

Underlined sequences have homology to the codon optimized OvHV-2 ORF8.
