# Supplementary material for: Ovine Herpesvirus 2 Glycoprotein B Complementation Restores Infectivity to a Bovine Herpesvirus 4 gB-Null Mutant
Source: Pathogens. 2024 Mar 1;13(3):219. doi: 10.3390/pathogens13030219 (PMC10974308; doi:10.3390/pathogens13030219)
Supplement: Supplementary file 1 [file pathogens-13-00219-s001.zip › pathogens-2819681-supplementary Table S2.pdf]

Table S2. Individual percentages of inhibition in AlHV-1ΔgB/OvHV-2-gB /OvHV-2-gB neutralization assay.

Rabbit ID	Group	Neutralizing antibodies			
		Plasma		BAL[a]	NS[a]
		0 DPI	64 DPI		
3060*	V	7.3	100.0	65.1	33.9
3061*	V	15.5	52.0	53.5	49.7
3062	V	3.2	26.4	32.8	36.9
3063	V	8.8	68.8	47.8	52.3
3064	V	11.4	29.1	35.1	47.8
3065	V	13.7	100.0	61.0	45.2
3066	V	2.4	42.6	49.3	35.4
**Avg (V)**		**9.4**	**59.8**	**49.2**	**43**
3070	M	9.5	0.0	21.9	34.3
3071	M	11.8	3.5	12.9	14.4
3072	M	15.5	6.5	41.8	22.7
3073	M	6.9	12.5	29.1	26.4
3074	M	0.5	5.0	12.5	26.8
3075	M	11.4	11.0	20.8	34.3
3076	M	9.9	7.3	17.0	43.3
**Avg (M)**		**8.9**	**6.5**	**22.3**	**28.9**

[a], Terminal samples; *, rabbits that were protected from MCF following challenge; V, BoHV-4ΔgB/OvHV-2-gB immunization; M, mock immunization; BAL, bronchoalveolar lavage; NS, nasal secretion; DPI, days post-prime immunization (64 DPI: OvHV-2 challenge, 85-139 DPI: terminal samples collected at necropsy).
